# The antidepressant fluoxetine induces necrosis by energy depletion and mitochondrial calcium overload

**DOI:** 10.18632/oncotarget.13689

**Published:** 2016-11-29

**Authors:** Emilie Charles, Mehdi Hammadi, Philippe Kischel, Vanessa Delcroix, Nicolas Demaurex, Cyril Castelbou, Anne-Marie Vacher, Anne Devin, Thomas Ducret, Paula Nunes, Pierre Vacher

**Affiliations:** ^1^ INSERM U1218, Institut Bergonié, Bordeaux, France; ^2^ Laboratory of Cellular and Molecular Physiology EA4667, Université de Picardie Jules Verne, SFR CAP-SANTE (FED 4231), Amiens, France; ^3^ Department of Cell Physiology and Metabolism, University of Geneva, Geneva, Switzerland; ^4^ Institut de Biochimie et Génétique Cellulaires, UMR 5095, Bordeaux, France; ^5^ INSERM U1045, Centre de Recherche Cardio-Thoracique, Bordeaux, France; ^6^ Université de Bordeaux, Bordeaux, France

**Keywords:** fluoxetine, CRAC, calcium overload, respiratory chain, cell death

## Abstract

Selective Serotonin Reuptake Inhibitor antidepressants, such as fluoxetine (Prozac), have been shown to induce cell death in cancer cells, paving the way for their potential use as cancer therapy. These compounds are able to increase cytosolic calcium concentration ([Ca^2+^]_cyt_), but the involved mechanisms and their physiological consequences are still not well understood. Here, we show that fluoxetine induces an increase in [Ca^2+^]_cyt_ by emptying the endoplasmic reticulum (ER) through the translocon, an ER Ca^2+^ leakage structure. Our data also show that fluoxetine inhibits oxygen consumption and lowers mitochondrial ATP. This latter is essential for Ca^2+^ reuptake into the ER, and we postulated therefore that the fluoxetine-induced decrease in mitochondrial ATP production results in the emptying of the ER, leading to capacitative calcium entry. Furthermore, Ca^2+^ quickly accumulated in the mitochondria, leading to mitochondrial Ca^2+^ overload and cell death. We found that fluoxetine could induce an early necrosis in human peripheral blood lymphocytes and Jurkat cells, and could also induce late apoptosis, especially in the tumor cell line. These results shed light on fluoxetine-induced cell death and its potential use in cancer treatment.

## INTRODUCTION

The antidepressant fluoxetine belongs to the Selective Serotonin Re-uptake Inhibitor (SSRI) family. SSRIs enable an increase in serotonin concentration in the synaptic cleft by preventing serotonin re-uptake back into the excitatory neuron [1]. SSRIs are effective antidepressants that possess an advantageous safety profile, especially concerning overdose. In most countries, fluoxetine (Prozac^®^) was the first SSRI that became available for clinical use [2]. Since then, fluoxetine has become one of the most widely used antidepressants.

In addition to their neurological effects, SSRIs – and especially fluoxetine – display toxic properties towards cancer cells, which are dissociated from their selective inhibition of serotonin reuptake. Indeed, fluoxetine has been shown to induce cell death in cancer cells *in vitro* [3–11] and to prevent the growth of tumors *in vivo* [5, 12–14]. Fluoxetine reduces cell viability in various models of cancer. Moreover, fluoxetine does not decrease the viability of non-cancer cell lines such as HSF [4] or primary cells such as peripheral blood mononuclear cells and B lymphocytes [9], suggesting that fluoxetine selectively kills tumor cells. Several types of cell death seem to be involved, with various publications reporting not only apoptosis [7–10], but also autophagy [10]. However, the precise mechanisms involved in fluoxetine-induced cell death remain largely unresolved at this time.

Fluoxetine and SSRIs also have reported effects on cytosolic calcium concentration ([Ca^2+^]_cyt_) and on ion channels, which can be either activated or inhibited. For example, numerous experiments have shown that fluoxetine induces an increase in [Ca^2+^]_cyt_ in immune cells [9, 10, 15–17] and central nervous system cells [18, 19]. This effect is found both in healthy and cancer cell models. Reports that fluoxetine induces Ca^2+^ release from the Endoplasmic Reticulum (ER) and mimics B-cell receptor (BCR) ligation [9, 20] suggest that the pathway could involve PhosphoLipase C (PLC) activation, leading to the production of Inositol 1,4,5-trisPhosphate (IP_3_) and to the activation of the IP_3_ Receptors (IP_3_R) located within the ER membrane. However, other authors have shown that IP_3_ is on the contrary not involved in fluoxetine-induced increase in [Ca^2+^]_cyt_ [16, 17, 20]. Nevertheless, whether IP_3_R is involved or not, Ca^2+^ is released from an intracellular compartment after a fluoxetine treatment [20]. It appears that the increase in [Ca^2+^]_cyt_ induced by fluoxetine is due to a Ca^2+^ entry [10, 15–17, 20]. Ca^2+^ is a second messenger, which is of utmost importance for numerous cellular processes including cell death. Hence, Ca^2+^ homeostasis is crucial, and it is well known that Ca^2+^ overload or an alteration in Ca^2+^ levels within different cellular compartments can be cytotoxic and may lead to cell death by necrosis, apoptosis or autophagy [21, 22]. Notably, mitochondria are a central compartment regarding Ca^2+^-induced cell death, and fluoxetine is found mainly accumulated in this organelle [23]. Overall, further insight is needed in order to elucidate the pathways involved in the increase in [Ca^2+^]_cyt_ triggered by fluoxetine.

The purpose of this study was thus to determine the signaling pathway triggered by fluoxetine, leading to a [Ca^2+^]_cyt_ increase in both cancer and healthy immune cells. For cancer cells, both adherent and non-adherent cell models were used. Additionally, we studied the relationship between the Ca^2+^ pathway and the cell death pathway. We confirmed that fluoxetine induces an ER-dependent cytosolic Ca^2+^ increase in adherent and non-adherent cell models. However, our data shows that this cytosolic Ca^2+^ increase is due to a “thapsigargin-like” effect, where Ca^2+^ leaves the ER via the translocon and triggers Store-Operated Ca^2+^ Entry (SOCE). The initial calcium leak is produced by a direct or indirect inhibition of SERCA activity, since fluoxetine impairs ATP production by inhibiting the respiratory chain. The rise in [Ca^2+^]_cyt_ resulted in a mitochondrial Ca^2+^ overload leading to cell death, mainly by necrosis.

## RESULTS

### Fluoxetine induces an increase in the cytosolic Ca^2+^ concentration resulting from Ca^2+^ release as well as Ca^2+^ entry

**Figure 1:**
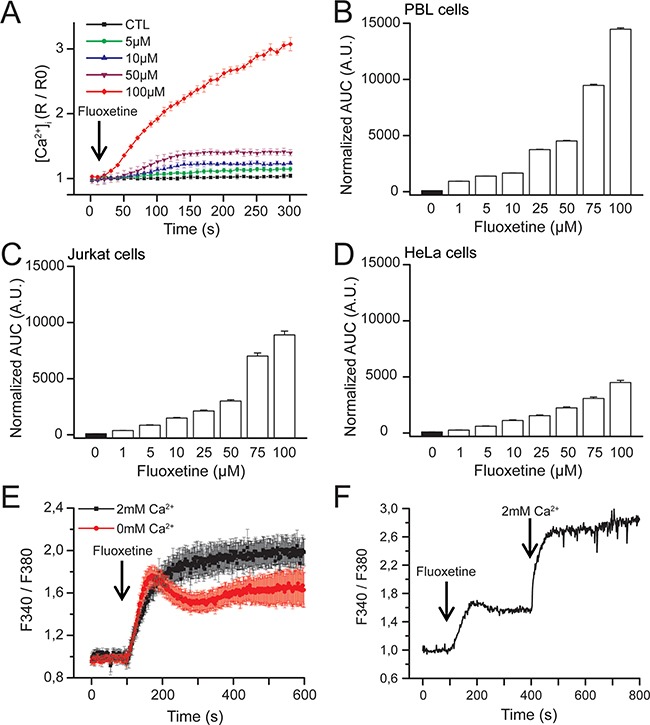
Dose-dependent effects of fluoxetine on [Ca2+]cyt **A**. Jurkat cells loaded with FuraPE3-AM Ca^2+^ probe were placed in a 2mM Ca^2+^ containing medium. Fluoxetine injection (5 to 100μM) is indicated by the arrow. Ratios were normalized to baseline. Results correspond to 3 independent experiments with 12 averaged wells per experiment. **B, C** and **D**. Quantification of Ca^2+^ responses induced by fluoxetine in a medium containing 2mM Ca^2+^ in PBL cells (B), Jurkat cells (C) and HeLa cells (D). The reported values represent the area under the curve (AUC), expressed in arbitrary units (A.U.). **E**. Jurkat cells were loaded with FuraPE3-AM, and fluorescence ratios were recorded in the presence (2mM, black trace) or absence (0mM, red trace) of external Ca^2+^. **F**. Jurkat cells loaded with Fura2AM were incubated first with fluoxetine (100μM) in a Ca^2+^-free medium. External Ca^2+^ was only added a few minutes later.

In Jurkat cells, fluoxetine can induce a dose-dependent increase in [Ca^2+^]_cyt_ (Figure 1A). In order to determine the effects of fluoxetine on [Ca^2+^]_cyt_ in PBLs cells and the adherent HeLa cancer cell line, we performed dose-response experiments using fluoxetine concentrations ranging between 1 and 100μM, and monitored Fura2-AM fluorescence immediately upon fluoxetine addition. Fluoxetine induces a dose-dependent increase in [Ca^2+^]_cyt_ in both PBLs (Figure 1B) and HeLa cells (Figure 1D). Jurkat cells are also shown for comparison under the same experimental settings in Figure 1C.

To determine whether this [Ca^2+^]_cyt_ increase results from an extracellular influx, from a mobilization of Ca^2+^ from the intracellular stores or both, experiments were carried out in the presence or absence of 2mM Ca^2+^ in the extracellular medium. As shown in Figure 1E for Jurkat cells, the addition of fluoxetine induces a sustained increase in [Ca^2+^]_cyt_ in the presence of extracellular Ca^2+^. In the absence of Ca^2+^, however, a fluoxetine addition leads to a smaller change in [Ca^2+^]_cyt_, this increase being transient, returning to the basal level after several minutes. The importance of extracellular Ca^2+^ was confirmed by another experiment in which cells were first placed in a Ca^2+^-free medium, and Ca^2+^ was perfused after fluoxetine addition (Figure 1F).

### Fluoxetine-induced Ca^2+^ entry involves CRAC channels

**Figure 2:**
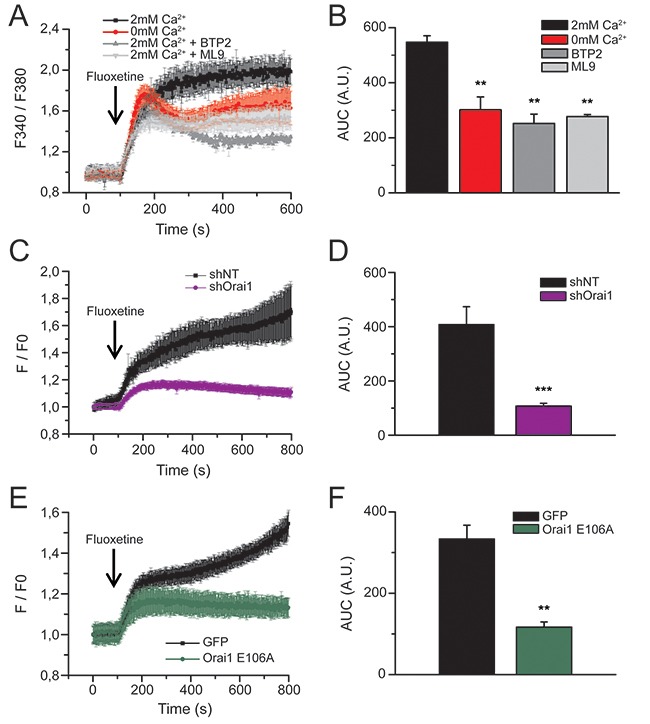
Fluoxetine-induced Ca2+ entry involves CRAC channels **A**. Jurkat cells were loaded with FuraPE3-AM, and fluorescence ratios were recorded in the presence (2mM, black trace) or in the absence (0mM, red trace) of external Ca^2+^. Fluoxetine injection (100μM) is indicated by the arrow. Inhibitors of Orai1 channels (BTP2, 10μM, dark grey trace) and Stim proteins (ML9, 10μM, light grey trace) were added in the presence of 2mM Ca^2+^. **B**. Quantification of results obtained in A: histogram bars represent the area under the curve (AUC), expressed in arbitrary units (A.U.), for experiments without external calcium (0mM Ca^2+^), with external calcium (2mM Ca^2+^), with BTP2 and with ML9. **C**. and **E**. Fluorescence ratios of Jurkat cells loaded with FuraPE3-AM, either transfected with a non-targeting shRNA (shNT) or a shRNA targeting Orai1 (shOrai1, C), or with a control GFP expression plasmid (GFP) and a plasmid allowing the expression of a non-conducting pore - dominant negative Orai1 (Orai1 E106A, E). Fluoxetine injection (100μM) is indicated by the arrow. **D** and **F**. Quantification of results obtained in C and E, respectively: histogram bars represent the area under the curve (AUC), expressed in arbitrary units (A.U.).

To determine how extracellular Ca^2+^ is transferred into cells upon fluoxetine treatment, we specifically studied Calcium Release-Activated Ca^2+^ (CRAC) channels, constituted of Orai1, a plasma membrane protein, and STIM1, a calcium sensor located in the ER membrane. When Jurkat cells (Figure 2A and 2B), PBLs (Supplementary Figure S1A and S1B) and HeLa cells (Supplementary Figure S1C and S1D) were pre-treated with BTP2 or ML-9 (inhibiting Orai1 and STIM1, respectively), the addition of fluoxetine in a Ca^2+^-containing medium led to a transient and highly reduced rise in [Ca^2+^]_cyt_ (grey traces in Figure 2A, Supplementary Figure S1A and S1C), quite similar to the effects observed in the absence of extracellular Ca^2+^ (red trace in Figure 2A). Furthermore, Jurkat cells expressing shOrai1 exhibited a less intense [Ca^2+^]_cyt_ increase (Figure 2C and 2D). Similar results were obtained with a non-conducting pore mutant of Orai1 (E106A), acting as a dominant negative (Figure 2E and 2F). These results clearly show that Store-Operated Ca^2+^ (SOC) channels are involved in fluoxetine-induced Ca^2+^ entry.

### Fluoxetine-induced Ca^2+^ release is independent of the PLC/IP_3_/IP_3_R pathway, RyR and of TCP

**Figure 3:**
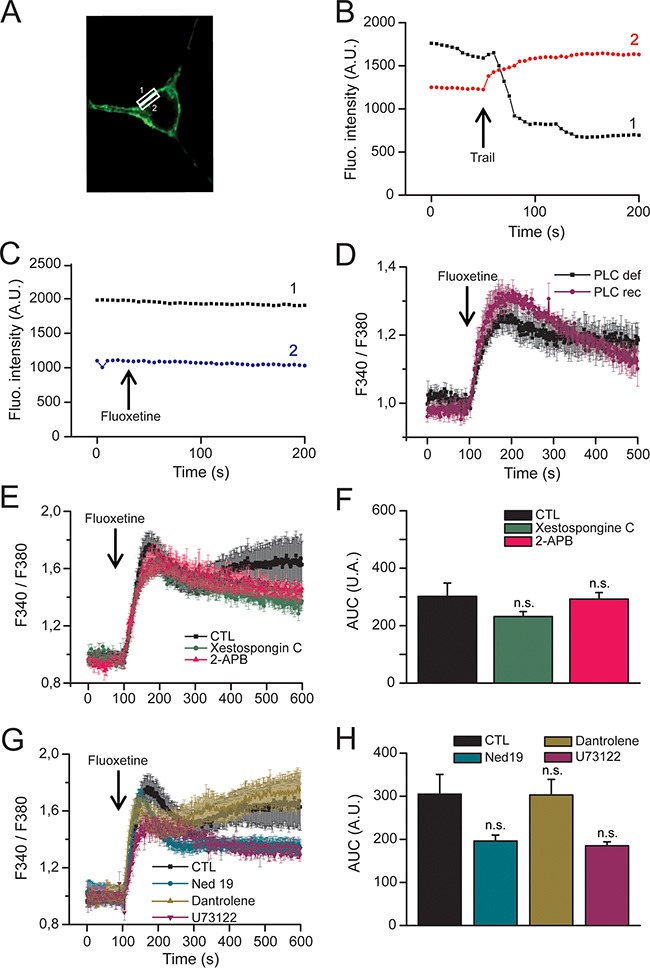
Fluoxetine does not induce hydrolysis of the membrane bound PIP2 **A**. Confocal microscopy images of HeLa cells, transfected with a construct enabling transient expression of a fusion protein [Pleckstrin Homology (PH) domain of PLC] / eGFP fluorescent protein, allowing to locate membrane PIP_2_, as well as cytosolic IP_3_. Fluorescence was measured in zones of interest delimited by rectangles, corresponding to membrane (rectangle “1”) and cytosolic regions (rectangle “2”), respectively. **B** and **C**. Time course of fluorescence emission, at the membrane level (black trace) and cytoplasm (red or blue trace): B - To induce IP_3_ translocation from membrane to cytoplasm, rhTRAIL (100ng/mL) was injected when indicated by arrow. C - Fluoxetine (100μM) does not induce IP_3_ translocation. **D**. Jurkat cells were loaded with FuraPE3-AM Ca^2+^ probe. Cells were either PLCγ deficient (PLC def) or reconstituted with a functional PLCγ (PLC rec). **E**. Jurkat cells were loaded with FuraPE3-AM, and fluorescence ratios were recorded in the presence of 2mM of external Ca^2+^ (black trace). Ratios were also recorded in the presence of the IP_3_Rs inhibitors xestospongin C (500nM) and 2-APB (44μM, green and red traces, respectively). **F**. Quantification of results obtained in E: histogram bars represent the area under the curve (AUC), expressed in arbitrary units (A.U.), for control (CTL), xestospongin C and 2-APB experiments. **G**. Jurkat cells were loaded with FuraPE3-AM, and fluorescence ratios were recorded in the presence of 10μM dantrolene (inhibitor of RyR1 and RyR3 isoforms, green trace), 10μM U73122 (inhibitor of PLCβ, purple trace) and 5μM Ned19 (TPC inhibitor, blue trace). **H**. Quantification of results obtained in G: histogram bars represent the area under the curve (AUC), expressed in arbitrary units (A.U.), for control (CTL), dantrolene, U73122 and Ned19 experiments.

The involvement of CRAC channels highly suggests that fluoxetine leads to a depletion of intracellular Ca^2+^ stores. In line with this, depletion of ER calcium with thapsigargin (TG) abolished fluoxetine-induced Ca^2+^ signals in HeLa cells (Supplementary Figure S2A). However, when cells were pre-treated with fluoxetine, TG-induced Ca^2+^ signals were also abolished (Supplementary Figure S2B). Several publications have shown that fluoxetine indeed induces Ca^2+^ release [9, 20], potentially in a PLC and IP_3_-independent manner [15, 17, 20]. To more thoroughly characterize the precise ER receptors or pumps responsible for the [Ca^2+^]_cyt_ increase, we used various pharmacological inhibitors and modified cell lines. First, we have ruled out the implication of the PLC/IP_3_ pathway in our cellular model by monitoring IP_3_ localization in cells after the addition of fluoxetine. For this purpose, we used a fusion protein made of the Pleckstrin Homology (PH) domain of PLCδ and eGFP, which is able to bind PIP_2_ and IP_3_ and hence allows the monitoring of IP_3_ localization after PIP_2_ cleavage, regardless of the PLC isoform (Figure 3A). As a positive control we used TRAIL, a death ligand able to induce the PLC/IP_3_ pathway, leading to an increase in [Ca^2+^]_cyt_. The results show that TRAIL induces relocation of the IP_3_ fluorescence from the membrane to the cytosol, revealing PIP_2_ cleavage and IP_3_ release (Figure 3B). In contrast, fluoxetine does not elicit such a relocation of the fluorescence (Figure 3C). Thus, we conclude that fluoxetine does not activate the PLC/IP_3_ pathway in order to trigger the increase in [Ca^2+^]_cyt_. Using PLCγ1^-/-^ Jurkat cells, we also show that PLCγ1 deficiency does not suppress fluoxetine-induced intracellular Ca^2+^ mobilization (Figure 3D). Moreover, upon treatment with two inhibitors of IP_3_Rs (Figure 3E and 3F), namely 2-APB (red trace) and xestospongin C (green trace), no significant difference concerning Ca^2+^ mobilization was revealed upon fluoxetine application. Finally, similar to B cells [20], other experiments using U73122, a pharmacological inhibitor of PLCβ [24], did not reveal significant difference in the [Ca^2+^]_cyt_ profile after fluoxetine treatment (Figure 3G and 3H). Together, these results demonstrate that the PLC/IP_3_/IP_3_R pathway is likely not involved in the fluoxetine-induced calcium release from the ER. Other receptors that could potentially have a role in the increase in [Ca^2+^]_cyt_ are the Ryanodine receptors (RyR). Using dantrolene (an inhibitor of RyR1 and RyR3 isoforms), we ruled out the potential implication of these receptors in fluoxetine-induced increase in [Ca^2+^]_cyt_ (Figure 3G and 3H). Finally, we ensured that Ca^2+^ was not released from lysosomes by using the Two Pore Channel (TPC) inhibitor, Ned19. Cells treated with Ned19 did not show significant modification of the fluoxetine-induced Ca^2+^ increase (Figure 3G and 3H).

### Fluoxetine induces ER calcium depletion through the translocon

**Figure 4:**
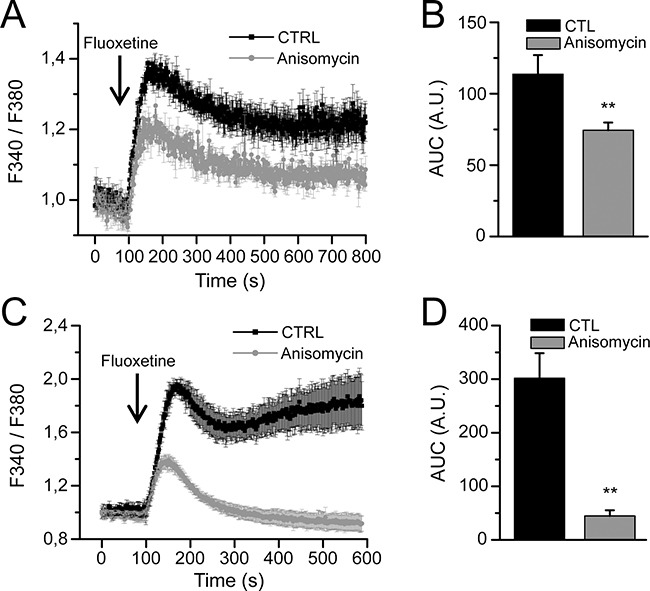
Fluoxetine induces ER Ca2+ depletion through the translocon Measurements of [Ca^2+^]_cyt_ in PBLs **A**. and in the Jurkat cell line **C**. cells were loaded with FuraPE3-AM, and fluorescence ratios were recorded either in control cells (CTL) or in cells pre-treated with anisomycin (30 min., 50μM), in absence of external Ca^2+^. **B** and **D**. Quantification of results obtained in A and C, respectively: histogram bars represent the area under the curve (AUC), expressed in arbitrary units (A.U.).

Since IP_3_R and RyR receptors, among others, are not likely to be involved in the ER Ca^2+^ release upon fluoxetine addition, we searched for another candidate. The translocon is an ER structure that enables the transfer of secretory proteins and lumenal domains of membrane proteins from the cytoplasm to the ER lumen [25]. However, it has also been shown that the translocon can mediate a Ca^2+^ leakage from the ER stores into the cytoplasm [26]. To determine whether this mechanism of calcium release could be involved in fluoxetine-induced [Ca^2+^]_cyt_ increase, we used a pharmacological inhibitor of the translocon, anisomycin (an inhibitor of peptidyl-transferase, leaving the translocon closed [26]). When PBL cells were pretreated 30 minutes with anisomycin, in the absence of extracellular Ca^2+^, the increase in [Ca^2+^]_cyt_ was reduced by 35% after fluoxetine addition in PBLs (Figure 4A and 4B). Interestingly, in Jurkat cells, pre-treatment with anisomycin (30 minutes) reduced the levels of fluoxetine-induced Ca^2+^ release by 85% (Figure 4C and 4D), confirming the role of the translocon in fluoxetine-induced [Ca^2+^]_cyt_ rise.

### Fluoxetine-induced increase in [Ca^2+^]_cyt_ leads to calcium accumulation in mitochondria

**Figure 5:**
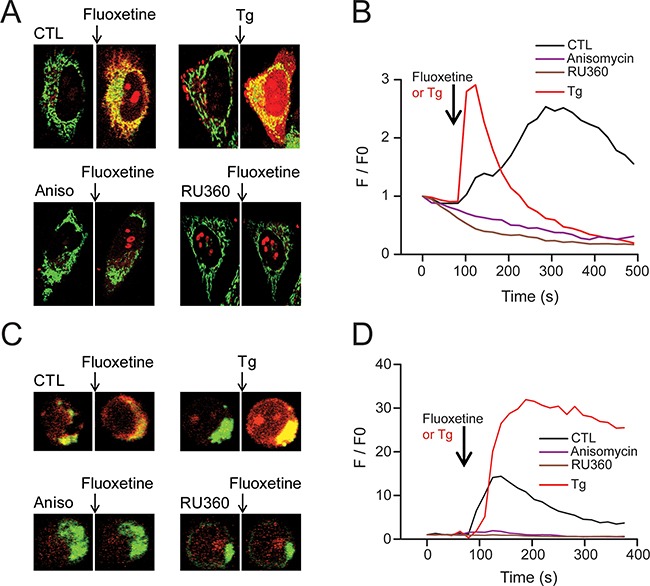
Fluoxetine leads to Ca2+ accumulation into mitochondria **A, C**. Confocal microscopy images obtained from HeLa cells (A) and PBL cells (C) loaded with the mitochondrial Ca^2+^ probe Rhod2-AM, as well as the mitochondrial probe MitoTracker^®^ green. The increase in mitochondrial Ca^2+^ load induced an increase in the red staining after fluoxetine or TG application, but not after pretreatment with an inhibitor of the translocon (anisomycin, 50μM) or an inhibitor of the MCU (RU360, 10μM). **B**. Rhod-2 fluorescence intensity of representative HeLa cells (as shown in ***A***) over time before and after addition of Tg or fluoxetine (either in control cells or in cells pre-treated with anisomycin or RU360). **D**. Rhod-2 fluorescence intensity of representative PBL cells (as shown in ***C***) over time before and after addition of Tg or fluoxetine (either in control cells or in cells pre-treated with anisomycin or RU360).

To decipher the downstream events of the [Ca^2+^]_cyt_ increase after a fluoxetine addition, we took a closer look at the mitochondria, in order to determine if Ca^2+^ could be stored in this compartment. For that purpose, we performed confocal imaging experiments with HeLa cells, using the mitochondrial marker and Ca^2+^ sensing probe Rhod2, in association with the Mitotracker Green mitochondrial probe. The addition of fluoxetine led to an increase in red fluorescence, with the overlap with the Mitotracker Green signal yielding a yellow color, demonstrating the increase in mitochondrial Ca^2+^ concentration ([Ca^2+^]_mt_, Figure 5A, CTL). For the sake of comparison, we also used TG, a compound that is well known for inhibiting SERCA pumps, raising thus [Ca^2+^]_cyt_ by ER depletion and Ca^2+^ entry through SOCE (Figure 5A, Tg). Tg induced a larger and faster, but more transient, Ca^2+^ entry in the mitochondria of HeLa cells (Figure 5B). Interestingly, we could not observe any increase in [Ca^2+^]_mt_ when cells were pretreated with the translocon inhibitor, anisomycin (Figure 5A and 5B). Similar results were obtained on PBLs cells (Figure 5C and 5D), demonstrating that a reduction of only 35% of the fluoxetine-induced cytosolic calcium increase (Figure 4A and 4B) was sufficient to completely abolish the fluoxetine-induced mitochondrial Ca^2+^ increase in these cells (Figure 5D). Next, to identify whether the Ca^2+^ uptake was mediated by the Mitochondrial Ca^2+^ Uniporter (MCU), we used Ru360, a specific inhibitor of the uniporter. We show that in the presence of Ru360, the fluoxetine-induced increase in [Ca^2+^]_mt_ was abolished in HeLa cells (Figure 5A and 5B), as well as in PBL (Figure 5C and 5D), suggesting the implication of the MCU in the fluoxetine-induced increase in [Ca^2+^]_mt_.

### Fluoxetine reduces oxygen consumption and leads to a drop in ATP content

**Figure 6:**
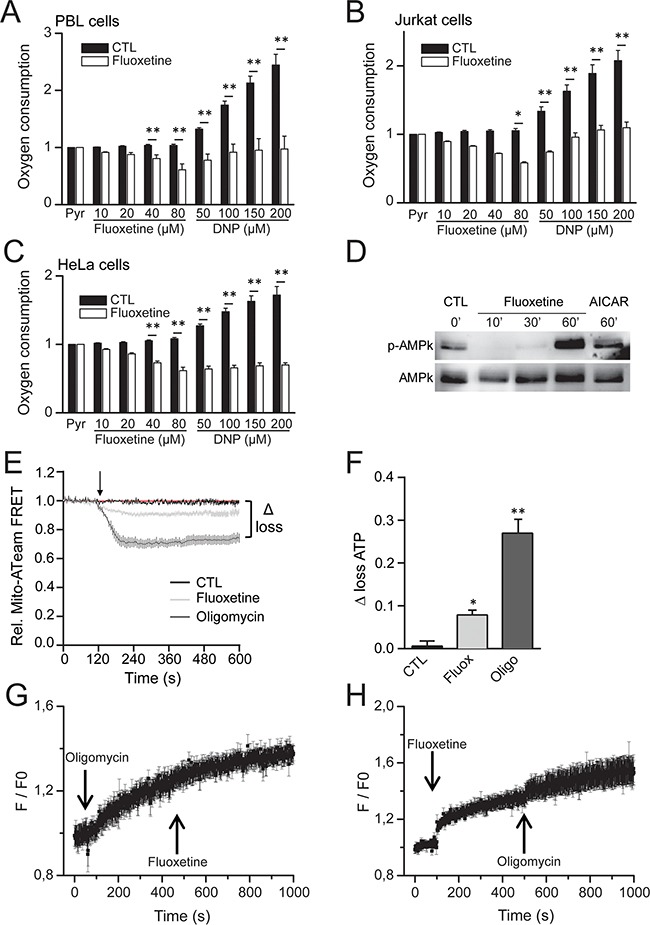
Fluoxetine blocks oxygen consumption and leads to a drop in ATP content **A, B** and **C**. Oxygen consumption rates were measured using an Oroboros oxygraph-2k, either without (CTL) or in the presence of fluoxetine or dinitrophenol (DNP, 50 to 200μM), in PBLs (A), Jurkat cells (B) or HeLa cells (C). All values were normalized *vs*. 5mM pyruvate. **D**. Western blotting showing the ratio AMPk / p-AMPk in Jurkat cells in control conditions (CTL, first lane) and in treated conditions, either with fluoxetine (50μM, second, third and fourth lanes, at 10, 30 and 60 minutes post-treatment, respectively) or with AICAR (an activator of AMP kinase, 0.65mM, fifth lane). **E** and **F**. Monitoring of ATP production via ATP imaging in single living cells using a Förster resonance energy transfer (FRET) - based fluorescent ATP probe (ATeam). Loss of ATP is visualized by a decrease in FRET (E), and quantified by the “Δ loss” value. The “Δ loss” values, obtained either with fluoxetine or the ATP synthase inhibitor oligomycin, calculated as the difference between initial and final normalized FRET signals levels after 10 min, are reported in F. **G**. Variations of [Ca^2+^]_cyt_ induced by fluoxetine (50μM) with oligomycin pre-treatment. **H**. Variations of [Ca^2+^]_cyt_ induced by 0.4μM oligomycin with fluoxetine pre-treatment. [Ca^2+^]_cyt_ was recorded in a whole cell population. Experiments shown in **G** and **H**. were performed in a Ca^2+^-free medium.

Since our results demonstrate that fluoxetine induces a mitochondrial Ca^2+^ overload, we next sought to determine if fluoxetine exerted other effects on mitochondria, for instance by addressing the potential involvement of the respiratory chain. We thus monitored oxygen consumption rate in different cell lines, and we showed that fluoxetine actually inhibits oxygen consumption in PBLs (Figure 6A), as well as in Jurkat cells (Figure 6B), and HeLa cells (Figure 6C), suggesting that fluoxetine inhibits the respiratory chain. When the ATPase activity was stimulated with dinitrophenol (DNP, Figure 6A, and 6C), fluoxetine was still able to exert an inhibitory effect. Since respiration is coupled to ATP production, we monitored the AMP-activated protein kinase (AMPk) activity by assessing the ratio p-AMPk / AMPk in Jurkat cells treated either with fluoxetine or with AICAR (an activator of AMP kinase). The AMPk system acts as a sensor of cellular energy status, and is activated by increases in the cellular AMP:ATP ratio caused by metabolic stresses that either interfere with ATP production or accelerate ATP consumption [27]. Figure 6D shows that fluoxetine is able to increase the ratio p-AMPk / AMPk, suggesting again that fluoxetine may be able to impair ATP production. We thus monitored ATP production via ATP imaging in single living cells using a Förster resonance energy transfer (FRET) - based fluorescent ATP probe, named ATeam [28]. We found that fluoxetine was able to reproducibly lower the steady-state levels of mitochondrial ATP (Figure 6E and 6F). Although the absolute decrease in ATP content in HeLa cells, which are highly glycolytic cells, was only on the order of 10%, it is noteworthy that this fluoxetine-induced decrease was equal to one third of the decrease observed using the same method with the potent ATP-synthase inhibitor oligomycin (Figure 6E and 6F). Thus, these observations are consistent with a model where fluoxetine partially decreases ATP synthesis by reducing respiratory chain activity. Additionally, we monitored the oligomycin and fluoxetine induced [Ca^2+^]_cyt_ changes (Figure 6G and 6H). When oligomycin was first added, a general [Ca^2+^]_cyt_ increase occurred due to ATP synthase inhibition, and fluoxetine was not able to further enhance [Ca^2+^]_cyt_ increase (Figure 6G). When fluoxetine was first added, the classical [Ca^2+^]_cyt_ increase occurred, and oligomycin was nonetheless able to raise slightly [Ca^2+^]_cyt_, most probably because of its more potent effect on ATP production (Figure 6H).

### Fluoxetine-induced Ca^2+^ signaling leads to late apoptosis and necrosis

**Figure 7:**
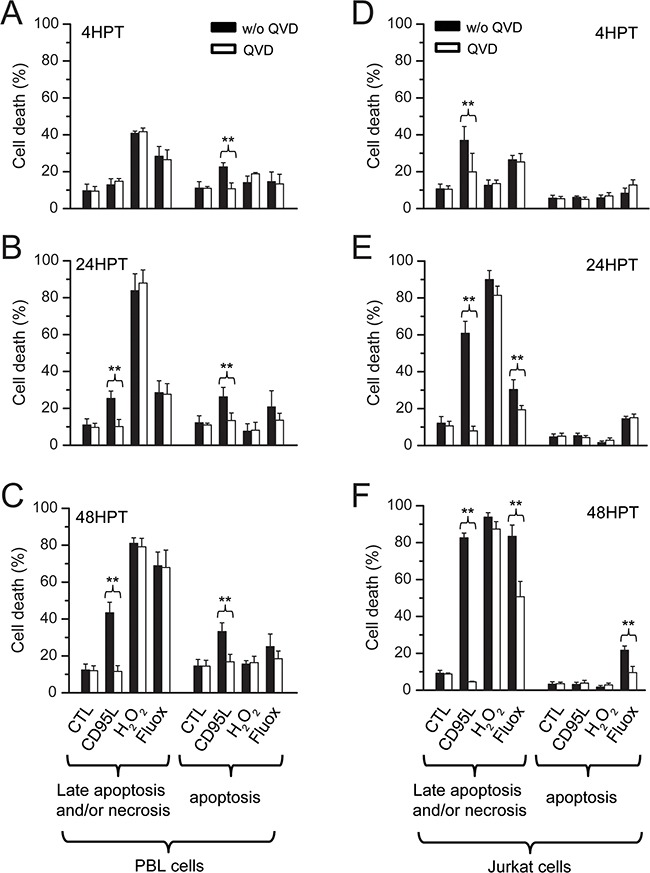
Fluoxetine-induced Ca2+ signaling leads to late apoptosis and/or necrosis Cell death was quantified in PBLs **A**, **B** and **C**. and in Jurkat cells **D**, **E** and **F**. using FACS. Analyses were conducted at 4h post-treatment (A and D), 24h post treatment (B and E) and 48h post treatment (C and F). Cell death was assessed by Annexin V (AV) and Propidium Iodide (PI) staining. Cells were cultured in normal conditions or treated with 20μM QVD, a broad spectrum caspase inhibitor. Cell death was induced with fluoxetine (40μM), but also with CD95L (2.5ng.ml^-1^ for Jurkat cells, 100ng.ml^-1^ for PBLs) and hydrogen peroxide (H_2_O_2_, 50μM). HPT: hours post-treatment.

We next sought to determine which mechanism was responsible for fluoxetine-induced cell death. Cell death was also induced by i) CD95L, a death ligand known to initiate the death-inducing signaling complex (DISC) upon binding to its receptor, that culminate in the induction of apoptosis [29]; ii) hydrogen peroxide (H_2_O_2_), one of the most biologically relevant member of the Reactive Oxygen Species (ROS [30]) generated in both intracellular and extracellular space in normal conditions, and also by phagocytic cells at sites of inflammation. High levels of ROS can cause necrosis, while lower levels can cause apoptosis [31]. H_2_O_2_ was indeed highly effective in inducing necrosis, especially at 24 and 48 hours post-treatment, in PBLs (Figure 7B and 7C) and Jurkat cells (Figure 7E and 7F). The 4h time frame was indeed not enough to see any hydrogen peroxide-induced necrosis on Jurkat cells (Figure 7D), whereas this compound was the most effective in inducing early necrosis in PBLs (Figure 7A). Fluoxetine was quite as effective in inducing necrosis in PBLs, with the largest effect seen at 48h (Figure 7C), and a less pronounced effect at 4h (Figure 7A) and 24h (Figure 7B). Jurkat cells followed apparently the same pattern, but the results were influenced by the application of QVD, a broad spectrum caspase inhibitor [32], suggesting that part of the cells positive for both annexin V (AV^+^) and propidium iodide (PI^+^) were late apoptotic cells (Figure 7E and 7F). CD95L also induced late apoptosis, especially in Jurkat cells (Figure 7E and 7F), but was less effective in PBLs (Figure 7A, and 7C). No necrosis was evidenced with CD95L since the QVD inhibitor indeed always abolished detection of AV^+^/PI^+^ cells. Early apoptosis could also be induced by CD95L in PBLs or by fluoxetine in Jurkat cells, but levels of apoptosis were far lower than necrosis / late apoptotic levels in the same conditions. This apoptosis was efficiently blocked by QVD, (Figure 7A, and 7F). H_2_O_2_ was clearly not a potent inducer of apoptosis. These results clearly show that late apoptosis and necrosis were the main mechanisms by which cell death occurred, in PBLs and Jurkat cells treated with fluoxetine (especially at 48h). The original Annexin V / PI plots are shown in Supplementary Figure S3.

## DISCUSSION

Although it is now widely accepted that SSRIs (such as fluoxetine) are able to induce a rise in intracellular Ca^2+^ and cell death, the exact underlying mechanisms remain obscure. Several groups agreed that fluoxetine releases Ca^2+^ from intracellular stores (including the recent work of Gobin and collaborators in human T lymphocytes [33]), in addition to stimulating extracellular Ca^2+^ entry [9, 20, 34, 35]]. In the present study, we confirm that fluoxetine indeed induces an increase in the cytosolic Ca^2+^ concentration, resulting from an intracellular ER Ca^2+^ release, followed by extracellular Ca^2+^ entry. Since this mechanism is reminiscent of capacitative channel activation, we further assessed the implication of Stim1 and Orai1. We found that these typical actors of store operated Ca^2+^ entry were involved in fluoxetine-induced [Ca^2+^]_cyt_ increase, in contrast to the recent finding in human T lymphocytes [33]. By using a variety of pharmacological tools and fluorescent assays, we show that the canonical PLC/IP_3_/IP_3_R pathway is not involved, in good agreement with other studies [15, 17, 20, 34]]. Our results suggest thus that fluoxetine effects are most likely not induced by G protein–coupled receptors signaling, nor through RyR or TPC. We were able to confirm reduced fluoxetine-induced Ca^2+^ signals following pre-treatment with agents depleting ER calcium, such as TG [9, 20] or ATP [34], as shown in Supplementary Figure S2A. However, it is noteworthy that fluoxetine, in turn, was also able to prevent TG from increasing the [Ca^2+^]_cyt_ in HeLa cells (Supplementary Figure S2B). This effect is not in agreement with what was found in human bladder cells [20], and the mechanism underlying these cell-type dependent differences remains to be determined. These results led us hypothesize that fluoxetine might have a “TG-like” effect on the ER. Upon TG application, this latter releases ER Ca^2+^ passively through leak channels, including the translocon [36]. In agreement with this hypothesis, the translocon blocker anisomycin either partially or strongly reduced fluoxetine-induced Ca^2+^ signals in PBL and Jurkat cells. How does fluoxetine induce a passive Ca^2+^ leak from the ER? One possibility is that it influences SERCA function, at least indirectly. Of note, the energy used for ER Ca^2+^ reuptake by SERCAs preferentially comes from ATP produced in mitochondria [37]. Since fluoxetine induced a decrease in respiration and a drop in mitochondrial ATP, it is conceivable that this could contribute to an inhibition of the Ca^2+^ reuptake back into the ER. Fluoxetine could be, in this case, considered as an indirect inhibitor of SERCAs. In line with this hypothesis, it has already been shown that the UCP3-mediated uncoupling of oxidative phosphorylation from ATP synthesis can modulate SERCA activity by decreasing mitochondrial ATP production [38].

**Figure 8:**
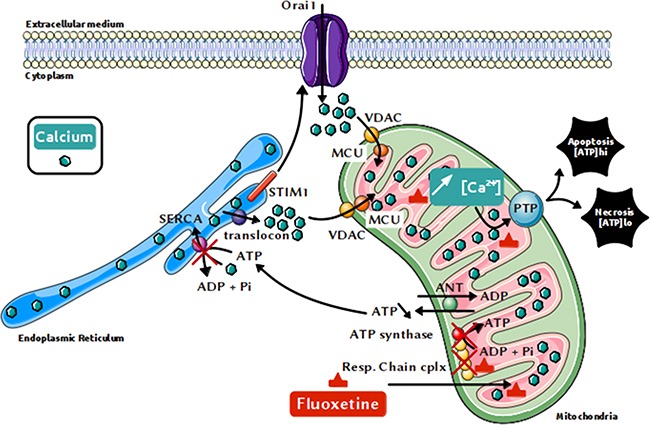
Proposed mechanism for the fluoxetine-induced increase in [Ca2+]cyt and cell death Fluoxetine, known to enter mitochondria, is likely able to inhibit directly the respiratory chain, which is necessary for mitochondrial ATP production, and thus for pumping calcium into the ER via SERCAs. Calcium leaks from the ER via the translocon, leading to ER calcium depletion, which in turn activates the store-operated calcium channels Orai1 via the reticular STIM1 protein. Once released from the endoplasmic reticulum, calcium quickly accumulates into the mitochondria, leading to mitochondrial calcium overload and cell death, mainly by necrosis.

Previous studies have shown that calcium leaking via the translocon triggers cell death, especially in epithelial cancer cells [39]. Our results demonstrate indeed that the fluoxetine-induced [Ca^2+^]_cyt_increase involving translocon correlates with cell death in PBLs and Jurkat cells. It is noteworthy that the Jurkat cell line was found to be sensitive to this fluoxetine-induced cell death, contrary to what Gordon's team reported [9]. Unlike TG, that is able to cause cell death mainly by apoptosis (with necrosis being involved to a lesser extent [40]), we found that fluoxetine-induced cell death was mainly caused by necrosis (and / or late apoptosis, especially in Jurkat cells). We hypothesized that these effects could be due to a mitochondrial Ca^2+^ overload, as recently shown in glioma cells [41]. It has already been demonstrated that fluoxetine preferentially accumulates into mitochondria [23], and most importantly, we show that fluoxetine induced Ca^2+^ accumulation into the mitochondria. At lower levels, Ca^2+^ can actually promote ATP synthesis, by stimulating the activity of various metabolic mitochondrial enzymes [42]. However, upon continuous Ca^2+^ overload, two mechanisms are engaged to eliminate Ca^2+^ excess from mitochondria: the NCLX (the mitochondria-specific sodium / calcium exchanger) and / or the PTP (Permeability Transition Pore) [43, 44]. The opening of this latter can cause the inner membrane of the mitochondria to depolarise [45], oxydative phosphorylations to stop (as evidenced herein), matrix swelling and membrane breaks, causing Ca^2+^ as well as proapoptotic molecules (such as cytochrome c) to leak out. Continuous mitochondrial permeability transition can lead to cell death by apoptosis or necrosis, depending on remaining cellular ATP. Intracellular ATP concentration appear indeed to be a key switch, pointing either to apoptosis or necrosis [46]. If the proportion of damaged mitochondria remains small, but sufficient to activate apoptosis, there will be enough ATP from intact mitochondria for apoptosis to occur [47]. If the proportion of damaged mitochondria is high, it is likely that signals emitted from damaged mitochondria will spread to all mitochondria [48], and subsequent ATP depletion will hinder assembly of the apoptotic machinery, ultimately leading to necrosis [46, 49]. Fluoxetine, at the concentration we used, may therefore induce directly necrosis and/or late apoptosis on most cells, and also apoptosis on a more resistant subpopulation of the cells. All results, as well as the proposed mechanism, are summarized in Figure 8. On the other hand, depletion of ER Ca^2+^ can also lead to ER stress, another pathway able to trigger cell death [50], especially when overstimulation occurs. Whether fluoxetine induces ER stress and whether this contributes to fluoxetine toxicity would require further study.

The cytotoxic fluoxetine effects are highly interesting for potential use in cancer therapies, particularly in light of beneficial psychological effects it may have on patients facing a life-threatening disease. The doses used in the present study to decipher the molecular mechanisms are admittedly high, but in line with most *in vitro* published studies, in which apoptotic effects of fluoxetine are seen from 0.1 to 100μM. Plasma concentrations found in patients treated with fluoxetine are usually in the low micromolar range: plasma concentrations at equilibrium in depressive-treated patients are rather comprised between 0.5 and 1.6μM, with high inter-individual variability [51]. After a 30-days treatment with a 40mg daily intake (neurologic disorders being treated within the 20 to 80mg range), plasma concentrations reach 1μM [52]. This concentration could conceivably retain significant apoptotic effects *in vivo*. Due to the relative safety of fluoxetine, dose escalation would also be an option: a study in which fluoxetine intakes ranged from 280 to 1260mg showed that plasma concentrations could reach 0.75 to 4.5μM (a study of 234 cases with fluoxetine overdose suggest that 1500mg intake is the upper limit [53]). Future investigation on the effects of fluoxetine concentrations within therapeutic range, as well as differential effects between normal and cancer cells (as already suggested by others [4, 9]), will both be necessary to assess whether and how fluoxetine may bring added value to existing cancer treatments in the clinic.

## MATERIALS AND METHODS

### Cell lines and PBLs

Jurkat cells and Jurkat variants (deficient for signaling proteins PLCγ1^-/-^) were obtained from Dr. Patrick Legembre (ER440-OSS CLCC Eugène Marquis, Université de Rennes 1). HeLa cells were bought from DSMZ. Peripheral blood mononuclear cells PBMC from healthy donors were isolated by Ficoll gradient centrifugation. Blood pouches were obtained from the EFS (Etablissement Français du Sang) Aquitaine-Limousin, after written informed consent was obtained from participants. Monocytes were depleted by a 2h adherence step, and the naive PBLs were stimulated as described previously [54]. HeLa and Jurkat T-leukemic cell lines were maintained in RPMI supplemented with 8% (v/v) heat-inactivated FCS and 2mM L-glutamine at 37°C in a 5% CO_2_ incubator.

### Pharmacological tools

Anisomycin, fluoxetine and ML9 were purchased from Sigma-Aldrich (L’Isle d’Abeau Chesnes, St-Quentin-Fallavier, France). RU360 were purchased from Santa Cruz Biotechnology (Heidelberg, Germany). Oligomycin and rhTRAIL were obtained from Merck Millipore (Fontenay sous Bois, France) and AdipoGen (Liestal, Switzerland), respectively. 2-APB, BAPTA-AM, BTP2, TG and xestospongin C were obtained from Calbiochem (Merck Chemicals Ltd., Nottingham, UK).

### Ca^2+^ monitoring

In cell populations, [Ca^2+^]_i_ was measured ratiometrically in Indo-1–loaded cells using a Hitachi F2500 spectrophotometer, as described previously [55]. Cells bathed in Hank's Balanced Salt Solution (HBSS) were placed in a quartz cuvette under continuous stirring. The Fura 2 fluorescence response to [Ca^2+^]_i_ was recorded as the F_340nm_/F_380nm_ fluorescence ratio. The values of the emitted fluorescence for each cell (F) were normalized to the starting fluorescence (F_0_) and reported as F/F_0_ (relative [Ca^2+^]_cyt_). Each experimental condition was repeated independently at least three times; values are reported as mean ± SD.

Single-cell [Ca^2+^]_i_ imaging was performed ratiometrically as described previously [54]. Cells were loaded with 5μM Fura2-PE3-AM for 30min at room temperature in (HBSS). Fura2-PE3-AM exhibits limited compartmentalization in intracellular stores and is leakage-resistant [56]. Imaging was controlled by Universal Imaging software, including MetaFluor and MetaMorph. All images were background-subtracted. Data processing was performed using OriginPro 7.5 software (OriginLab).

In some experiments, cells were placed in a Ca^2+^-free medium, consisting of the HBSS described above in which CaCl_2_ was omitted and 100μM EGTA was added in order to chelate residual Ca^2+^ ions. This medium was added to the cells just before recording to avoid intracellular calcium stores leaking.

### Mitochondrial calcium imaging

Jurkat, PBLs and HeLa cells were loaded with Rhod-2 AM fluorescent probe (1μM, 2h, and 37°C). Cells were grown on glass coverslips and placed in an Attofluor observation chamber for confocal microscopy (Zeiss LSM 510 Meta, with Planapochromat x63 oil immersion objective). Helium/Neon laser (543 nm) was used for excitation, and emitted fluorescence was recorded through a low-pass filter (560nm). Analysis was made with Zeiss software (Axiovision). Mitochondria were stained using Mitotracker green (200nM, 2h, and 37°C).

### Real time membrane PIP_2_ and cytosolic IP_3_ imaging

A fusion protein between PH domain (Pleckstrin Homology domain) of PLCγ1 and GFP (GFP-PH) was used. This PH domain has a high selectivity for PIP_2_ and a higher affinity for IP_3_ than for PIP_2_. It translocates thus from membrane to cytosol with IP_3_. This system enables thus real time IP_3_ production in living cells, with migration of green fluorescence (GFP) from the membrane to cytoplasm [57]. Construct was obtained from Pr. T. Meyer (Stanford University, CA, USA). HeLa cells were transfected with the Exgen500 kit (Euromedex) for plasmid expression.

### Western blot

Cells were lysed into lysis buffer containing 1% Na deoxycholate (Sigma), 150mM NaCl, 10mM PO_4_Na_2_/K, pH 7.2 and supplemented with inhibitor cocktail (Sigma), 2mM EDTA, 1mM sodium orthovanadate (New England Biolabs), phosphatase inhibitor (Thermo Fisher) and 5mM PMSF (phenylmethanesulfonyl fluoride, Sigma). Benzonase nuclease (Santa Cruz) was added to reduce viscosity in protein extracts. Protein content was measured using BCA method (Biorad). Each sample was dissolved in Laemmli 5X buffer, and then denatured at 100°C for 10min. After SDS-PAGE and transfer onto nitrocellulose membrane (Hybond, GE Healthcare), membranes were incubated with goat polyclonal primary antibodies against p-AMPk (1/1000, Millipore) or AMPk (1/1000, Sigma) overnight at 4°C. Membranes were incubated with anti-goat peroxidase-conjugated anti-IgG secondary antibody (1/5000, Santa Cruz), developed using ECL substrate solution, exposed to the Fusion Fx7 (Thermo Fisher) and analyzed using Quantity One software (Biorad).

### Respiration experiments

Respiration assays were performed using an Oroboros Oxygraph-2k. The oxygen consumption was measured polarographically at 37°C using a Clark oxygen electrode in a thermostatically controlled chamber. Respiratory rates (JO_2_) were determined from the slope of a plot of O_2_ concentration *versus* time, and normalized by million cells. Respiration assays were performed in the growth medium supplemented with HEPES 10mM, pH 7.2.

### ATP measurements

Mitochondrial ATP levels were measured by transfecting HeLa cells with the mitochondrially targeted ATP-sensitive FRET-based probe (Mito-ATeam), as previously described [38]. Briefly, cells seeded on coverslips were washed in a modified Ringer's medium (in mM: 140 NaCl, 5 KCl, 1 MgCl_2_, 2 CaCl_2_, 10 HEPES, and 10 glucose, pH 7.3) and images were collected using 440nm excitation and alternate 485/535 nm emission on an Axiovert S100 TV microscope through a ×40, 1.3 NA oil-immersion objective (Carl Zeiss AG, Switzerland) equipped with a 16-bit CCD camera, Xenon lamp and filter-based wavelength switcher (Visitron Systems GmbH, Germany). Minimum FRET emission ratios were obtained by washing cells in Ringer's were glucose was replaced by 2-deoxyglucose and contained 10μg/ml oligomycin. Relative changes in mitochondrial ATP are reported as background-subtracted FRET ratios normalized to baseline and minimum FRET emission ratios where Relative Mito-ATeam FRET = ((F_335_/_485_ - F_min_) / F_baseline_). The loss of ATP is reported as Loss ATP = (1 - Relative Mito-ATeam FRET) at t=10min after stimulation. Bars show mean ± SEM of 3 independent experiments.

### Cell death experiments

Cells were processed using annexin V – propidium-iodide (PI) apoptosis assay kit according to manufacturer's protocol (Life Technologies). A total of 10000 events were analyzed by flow cytometry.

### Statistics

The results are expressed as the mean ± standard error of the mean (SEM) of the indicated number of experiments (N=passage number, n=cell number). Statistical analysis was performed using the Student's t-test, a non-parametric Mann–Whitney test, or an ANOVA statistical test (Sigmastat). Differences between the values were considered significant when p < 0.05. * and ** mean p-values smaller than 0.05 and 0.01, respectively.

## SUPPLEMENTARY MATERIALS FIGURES



## Abbreviations

AICAR: 5-aminoimidazole-4-carboxamide-1-β-D-ribofuranoside
CRAC channels: Calcium Release-Activated Calcium channels
ER: Endoplasmic Reticulum
IP_3_: Inositol 1,4,5-tris-Phosphate
IP_3_R: IP_3_ Receptors
MCU: Mitochondrial Calcium Uniporter
PLC: PhosphoLipase C
PTP: Permeability Transition Pore
SERCA: Sarcoplasmic/Endoplasmic Reticulum Ca^2+^-ATPase
SOC: Store-Operated Channel
SOCE: Store-Operated Calcium Entry
SSRI: Selective Serotonin Re-uptake Inhibitor.
