# Supplementary material for: The antidepressant fluoxetine induces necrosis by energy depletion and mitochondrial calcium overload
Source: Oncotarget. 2016 Nov 29;8(2):3181–96. doi: 10.18632/oncotarget.13689 (PMC5356874; doi:10.18632/oncotarget.13689)
Supplement: Supplementary file 1 [file oncotarget-08-3181-s001.pdf]

The antidepressant fluoxetine induces necrosis by energy depletion and mitochondrial calcium overload

SUPPLEMENTARY FIGURES

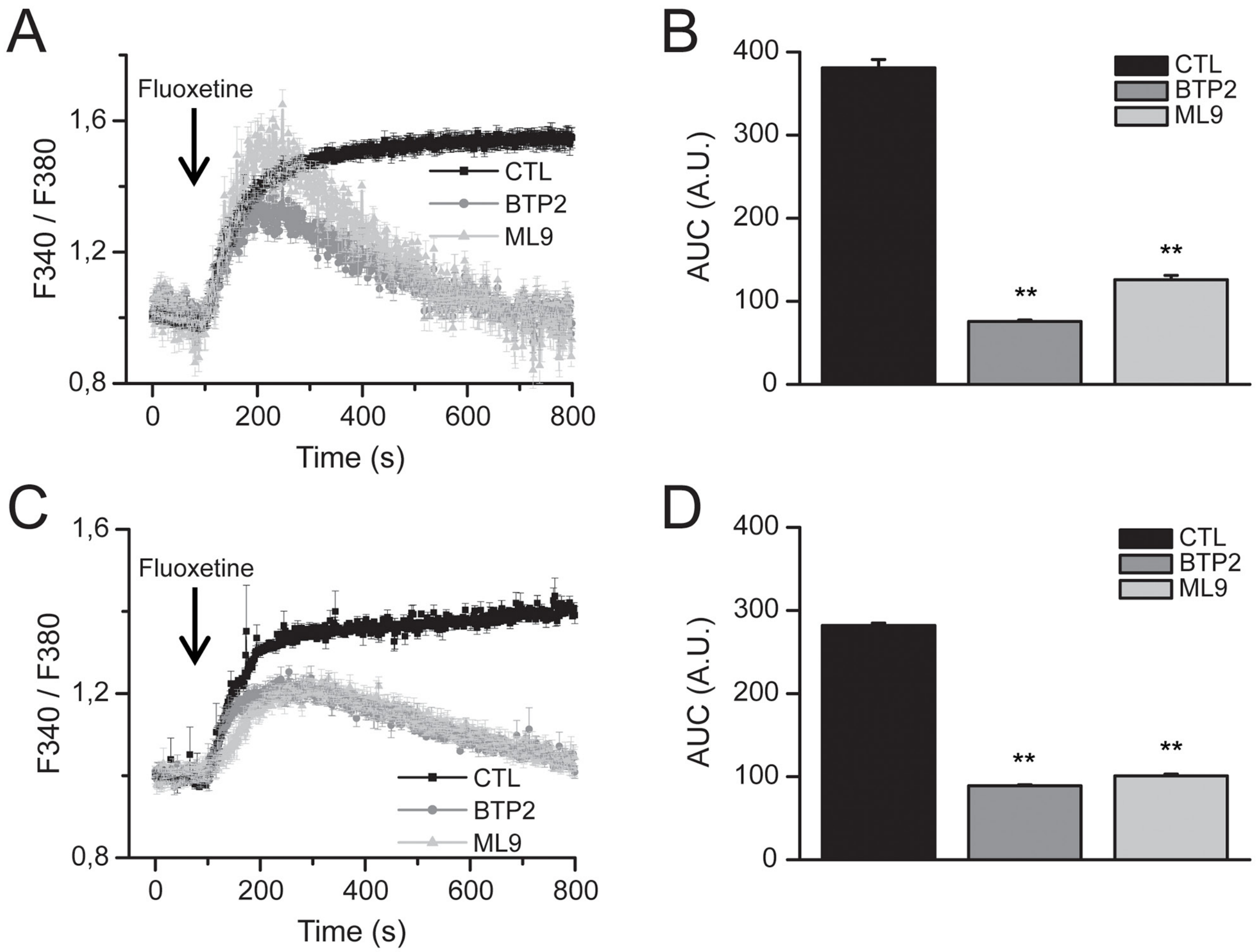

Supplementary Figure S1: Fluoxetine-induced Ca^{2+} entry involves CRAC channels. PBL cells **A.** and HeLa cells **C.** were loaded with FuraPE3-AM and fluorescence ratios were recorded in the presence of 2mM of external Ca^{2+} (black trace). Ratios were also recorded in the presence of the Orai1 inhibitor BTP2 (10µM, dark grey trace) and the Stim protein inhibitor ML9 (10µM, light grey trace). **B** and **D**. show the quantification of results obtained in A and B, respectively: histogram bars represent the area under the curve (AUC), expressed in arbitrary units (A.U.), for control (CTL), BTP2 and ML9 experiments.

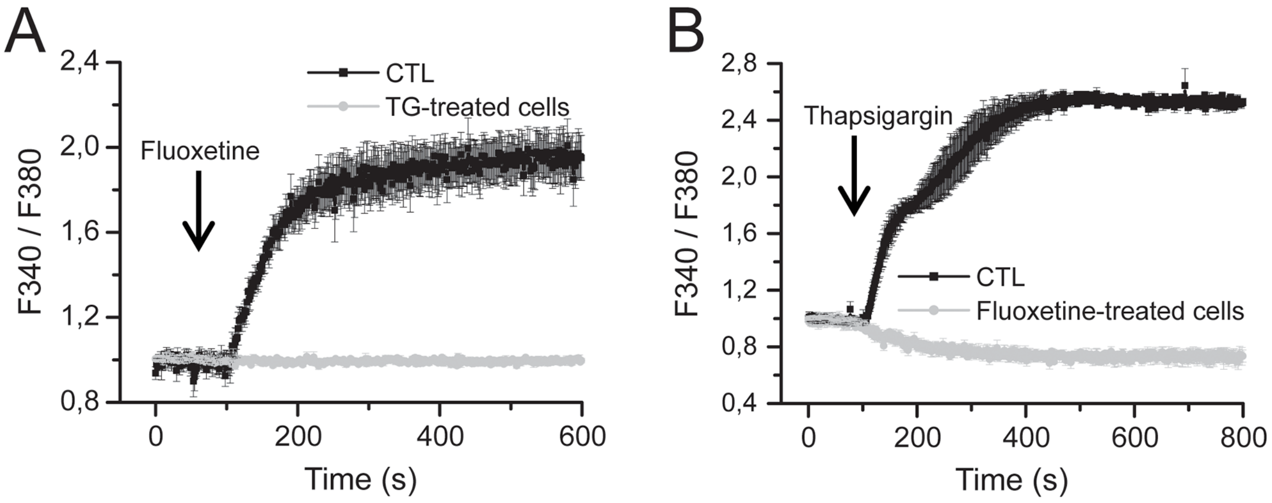

Supplementary Figure S2: Effectsof fluoxetine and TG pretreatments on $[Ca^{2+}]_{cyt}$ variations induced by TG and fluoxetine in HeLa cells. A. Variations of $[Ca^{2+}]_{cyt}$ induced by fluoxetine without (CTL, black trace) or with TG pre-treatment (light grey trace). **B.** Variations of $[Ca^{2+}]_{cyt}$ induced by TG without (CTL, black trace) or with fluoxetine pre-treatment (light grey trace).

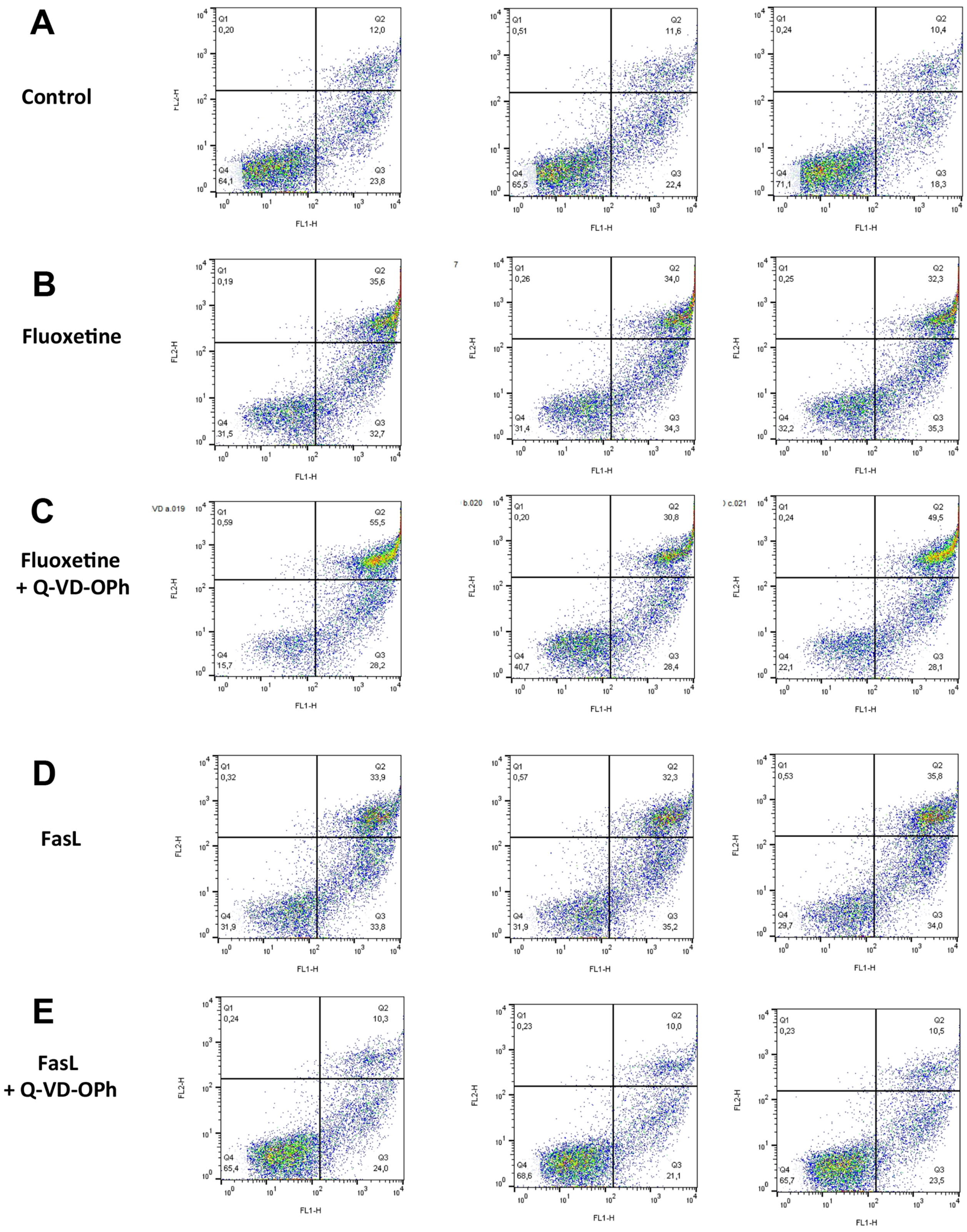

Supplementary Figure S3: Effects of fluoxetine on cell death in PBL cells. Dot-plot of Annexin V / Propidium iodide staining of PBL cells in control conditions **A.** in cells treated for 48h with fluoxetine (40µM, **B.**) fluoxetine (40µM) + Q-VD-Oph (20µM, **C.**) FasL (100ng.mL^{-1}, **D.**) FasL (100ng.mL^{-1}) + Q-VD-Oph (20µM, **E.**) (*Continued*)

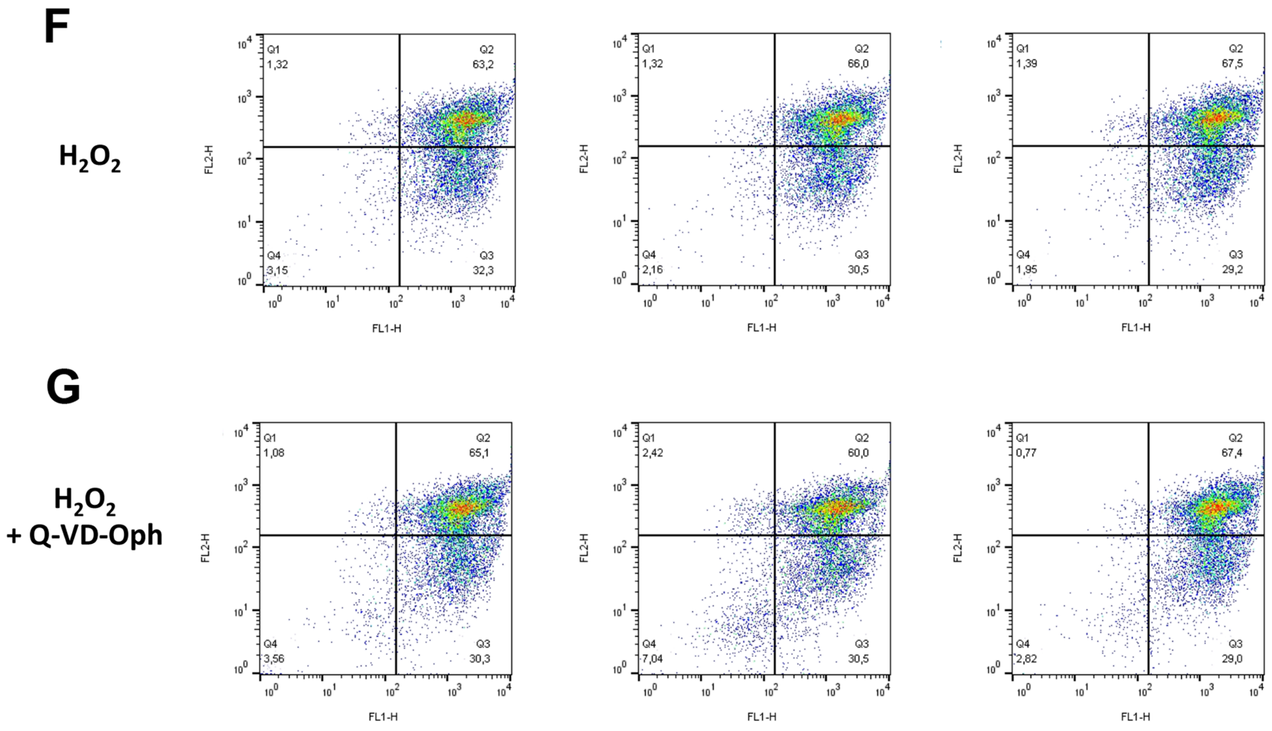

Supplementary Figure S3: Effects of fluoxetine on cell death in PBL cells. (***Continued***) H_20_2 (50µM, **F.**), H_20_2 (50µM) + Q-VD-Oph (20µM, **G.**)
